# Anticancer drugs and cardiotoxicity: the role of cardiomyocyte and non-cardiomyocyte cells

**DOI:** 10.3389/fcvm.2024.1372817

**Published:** 2024-07-11

**Authors:** Chrysa Koukorava, Katie Ahmed, Shrouq Almaghrabi, Amy Pointon, Malcolm Haddrick, Michael J. Cross

**Affiliations:** ^1^ Department of Pharmacology and Therapeutics, Institute of Systems, Molecular and Integrative Biology, University of Liverpool, Liverpool, United Kingdom; ^2^ Department of Life Sciences, Faculty of Science and Engineering, Manchester Metropolitan University, Manchester, United Kingdom; ^3^ Safety Sciences, Clinical Pharmacology and Safety Sciences, BioPharmaceuticals R&D, AstraZeneca, Cambridge, United Kingdom; ^4^ Medicines Discovery Catapult, Macclesfield, United Kingdom; ^5^ Liverpool Centre for Cardiovascular Science, Liverpool, United Kingdom

**Keywords:** cardiotoxicity, cardio-oncology, cardiomyocytes, endothelial, fibroblast

## Abstract

Cardiotoxicity can be defined as “chemically induced heart disease”, which can occur with many different drug classes treating a range of diseases. It is the primary cause of drug attrition during pre-clinical development and withdrawal from the market. Drug induced cardiovascular toxicity can result from both functional effects with alteration of the contractile and electrical regulation in the heart and structural changes with morphological changes to cardiomyocytes and other cardiac cells. These adverse effects result in conditions such as arrhythmia or a more serious reduction in left ventricular ejection fraction (LVEF), which can lead to heart failure and death. Anticancer drugs can adversely affect cardiomyocyte function as well as cardiac fibroblasts and cardiac endothelial cells, interfering in autocrine and paracrine signalling between these cell types and ultimately altering cardiac cellular homeostasis. This review aims to highlight potential toxicity mechanisms involving cardiomyocytes and non-cardiomyocyte cells by first introducing the physiological roles of these cells within the myocardium and secondly, identifying the physiological pathways perturbed by anticancer drugs in these cells.

## 1 Introduction

Cardiovascular toxicity can be defined as “chemically induced heart disease”. Drug discovery and development has improved significantly over recent years, particularly with respect to cancer therapeutics, altering the landscape for modern cancer therapy. The established chemotherapy drugs, developed in the 1950s–1970s, have been reinforced with the advent of molecular targeted cancer therapy and the recent development of immune checkpoint therapy providing an unprecedented arsenal of anti-cancer therapies. However, as cancer patients survive longer, often following treatment with multiple anticancer drugs for relatively longer periods of time, drug-induced organ toxicity, and in particular cardiotoxicity, is now becoming a major problem (1). Combined with an aging population, often with pre-existing cardiovascular problems, the cardiac safety of anticancer drugs is now necessitating the clinical implementation of the new field of cardio-oncology to manage and moderate cardiovascular toxicity (2, 3).

Ultimately the problematic endpoint of drug-induced cardiotoxicity is the inability of the heart to efficiently pump blood around the body. The specific mechanisms of drug-induced cardiovascular toxicities are not well understood and can occur via direct interactions of the drug with cardiomyocytes or indirectly through interactions with other components of the cardiovascular system. Direct actions can be functional or structural changes to the cardiomyocytes. Functional changes include inducing arrhythmias or QT prolongation by dysregulating cardiomyocyte contractility (4), while structural changes, such as cardiomyocyte hypertrophy or loss of viability can occur, which lead to an eventual decline in cardiac function. Drugs may also directly affect non-cardiomyocyte cells such as fibroblasts, macrophages and vascular muscle cells, adversely affecting their physiology (5, 6). Evidence suggests interactions between these cell types are essential to the metabolism, growth, contractile performance and rhythmicity of the myocardium.

## 2 Cardiac development and cardiac cell physiology

**Figure 1:**
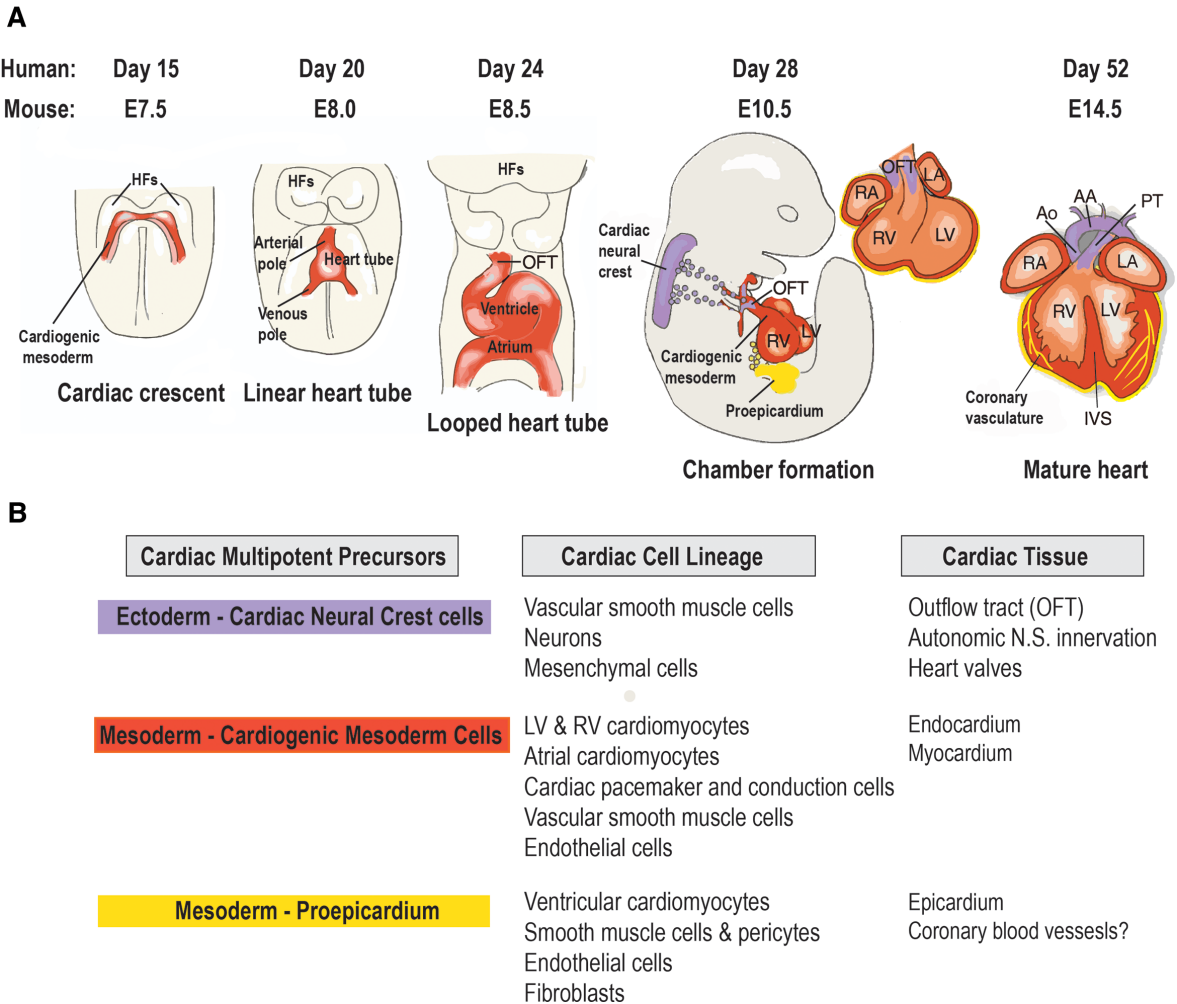
Origin and lineage relationship of cardiac cell types. (**A**) Contribution of three embryonic heart progenitor populations—cardiogenic mesoderm (red), cardiac neural crest (purple), and proepicardial organ (yellow)—to various heart compartments during cardiac morphogenesis. Cardiogenic mesoderm progenitors first appear beneath the head folds (HFs) at embryonic day 7.5 (E7.5) in the mouse embryo, then migrate ventrally to the midline (ML), forming the linear heart tube, which subsequently develops into the four heart chambers. Following heart tube looping (E8.5), cardiac neural crest progenitors migrate from the dorsal neural tube to the aortic arch arteries, differentiating into vascular smooth muscle cells of the outflow tract (OFT) by E10.5. Concurrently, proepicardial organ precursors contact the heart's surface, forming the epicardial mantle and later contributing to the coronary vasculature. By foetal stage E14, the heart chambers undergo septation and establish connections to the pulmonary trunk (PT) and aorta (Ao). (**B**) Cardiac cell types arising from the lineage diversification of the three embryonic precursor pools in the mouse heart. AA, aortic arch; IVS, interventricular septum; LA, left atrium; LV, left ventricle; RA, right atrium; RV, right ventricle. Adapted with permission from (10).

The cardiovascular system is the first functional organ to develop during human embryonic development (7). During gastrulation, a single-layered blastula is re-organized into three germ layers: a dorsal ectoderm, a ventral endoderm and a mesoderm layer. From the mesoderm germ layer myocardial progenitor cells differentiate into cardiomyocytes and non-cardiomyocytes (endothelial cells, postnatal cardiac progenitor cells and vascular smooth muscle cells) (8). These myocardial progenitor cells migrate to form a bilateral cardiogenic plate composed of two endocardial tubes. These tubes fuse to form a primary heart tube (9). At the cellular level the endocardial tubes originate from the mesoderm germ layer, which during cardiac embryogenesis differentiates into mesothelium, endothelium and myocardium enabling the development of both cardiomyocytes and non-cardiomyocytes (Figure 1). The primary heart tube consists of an inner endocardial layer and an outer myocardial layer separated by an extracellular matrix (11). This tubular heart further differentiates via folding and looping into the truncus arteriosus, bulbus cordis, primitive ventricle, primitive atrium and the sinus venosus forming a primitive heart containing the origins of cardiomyocytes and non-cardiomyocytes within different morphological arrangements (12). This primitive heart continues to elongate and loop anatomically arranging the cell populations into chambers and major vessels. This results in the aorta and pulmonary trunk originating from the truncus arteriosus, the right ventricle from the bulbus cordis, the left ventricle from the primitive ventricle, the left and right atria from the primitive atrium and SA node and coronary sinus from the sinus venosus. Further septa and valves develop separating the left and right sides of the heart and the major vessels from the heart, respectively, in conjunction cells of cardiac neural crest and proepicardium origin migrate. This cell migration enables the separation of outflow tracts and formation of the epicardium. The proepicardium cells are also the origin for coronary vasculature cells and cardiac fibroblasts (8). However, recent data have suggested that the coronary vasculature may be derived from multiple sources such as the proepicardium, the sinus venosus and ventricular endocardium and circulating endothelial progenitor cells (13). Ultimately, these developmental processes result in the formation of the myocardium consisting of the heart, coronary vascular and microvascular network. The developed heart is comprised of 30% cardiomyocytes and 70% non-cardiomyocyte cells including cardiac fibroblasts, endothelial cells and pericytes (14). Both endothelial cells and pericytes form a microvascular network surrounded by an extracellular matrix known as intramyocardial capillaries. This extensive microvascular network enables efficient perfusion of the heart delivering nutrients such as free-fatty acids and oxygen and facilitates cellular signaling (14).

Cardiomyocytes are the contractile competent muscle cells responsible for the coordinated contractile force required to eject blood from the left and right ventricles during systole (15). During pre-natal heart growth, cardiomyocytes show a high proliferation rate (16). After birth, the cardiomyocyte proliferation rate declines rapidly and remains at ∼1% in adult humans (17). This decline in proliferation is caused by cells exiting the cell cycle before cytokinesis causing cardiomyocytes to become binucleated and polyploid (18). The subsequent postnatal increase in cardiac volume occurs almost exclusively by hypertrophy of cardiomyocytes (19). Both binucleated and polyploid cardiomyocytes are generally believed to be terminally differentiated and unable to contribute to cardiomyocyte renewal during cardiac homeostasis and injury, which has profound implications for drug-induced cardiotoxicity. In contrast, the small remaining population of diploid, mononucleated cardiomyocytes is thought to still have the capacity to proliferate (20–22).

The cardiac microvascular endothelial cells are present both within the endocardium that forms the inner lining of the heart chambers and in intramyocardial capillaries. Their primary function is to supply cardiomyocytes with free-fatty acids and oxygen to meet their high metabolic demands. Other roles of cardiac endothelial cells include aiding the adherence of immune cells, allowing inter-cellular signalling factors to be communicated and to impede thrombus formation. Recent evidence suggests that cardiac microvascular endothelial cells are present at a higher abundance than previously reported, and have been estimated to be the most prominent cell type in the myocardium accounting for 40% of the cells (23). Cardiomyocyte-endothelial cell cross talk results in paracrine signalling to coordinate cellular responses within the myocardium. Cardiomyocytes secrete VEGF-A and angiopoetin-1 (Ang-1) which support angiogenesis and vessel maturation respectively (24). Cardiac endothelial cells are known to secrete proteins such as neuregulin-1 (NRG-1), which binds to and activates the receptor tyrosine kinase ErbB4 (HER4) on cardiomyocytes, allowing dimerization with ErbB2 (HER2) (25). This activates an intracellular signalling cascade in cardiomyocytes that suppresses apoptosis and stimulates a cardioprotective effect (24, 26).

Pericytes are also present in these cardiac capillaries, on average each endothelial cell is associated with two to three pericytes (27). These pericytes are contractile cells which wrap themselves around the microvasculature and support the normal functioning of the endothelial cells. They play many physiological roles from barrier function, regulation of homeostasis, facilitation of angiogenesis to initiation of coagulation (28).

Cardiac fibroblasts (CFs) are spindle shaped connective tissue cells that do not form a basement membrane but are responsible for the majority of the extracellular matrix (ECM) proteins found in the myocardium. During embryonic development, CFs arise from multiple sources. The epicardium is considered the major source of CFs found in the ventricular myocardium (29), with epicardial cells undergoing an epithelial-to-mesenchymal transition requiring expression of Tcf21 to generate CFs (30). Another significant population of CFs is derived from the endocardium at the time of endocardial cushion formation by an endothelial-to-mesenchymal transition (29, 31) and endocardial cells are generated in part from the second heart field progenitors (SHFPs). Lastly, a small fraction of fibroblasts are derived from neural crest lineages (32). Situated between cardiomyocytes, their close proximity teamed with the capacity to influence ECM composition provides fibroblasts with the ability to influence the phenotype of cardiomyocytes (33). Cardiac fibroblasts are essential for proper electrical conduction in the heart as they regulate calcium homeostasis through myocyte:fibroblast coupling (34, 35). In addition, cardiac fibroblasts help maintain tissue structure by regulating the deposition and remodelling of collagen and other ECM components (36, 37). Beyond their important role in maintaining normal myocardial function, CFs also contribute to adverse cardiac remodelling during pathological conditions, such as hypertension, myocardial infarction, and heart failure (38) and are involved in arrhythmia initiation and maintenance by affecting electrical propagation (39).

## 3 Cardiotoxicity

**Figure 2:**
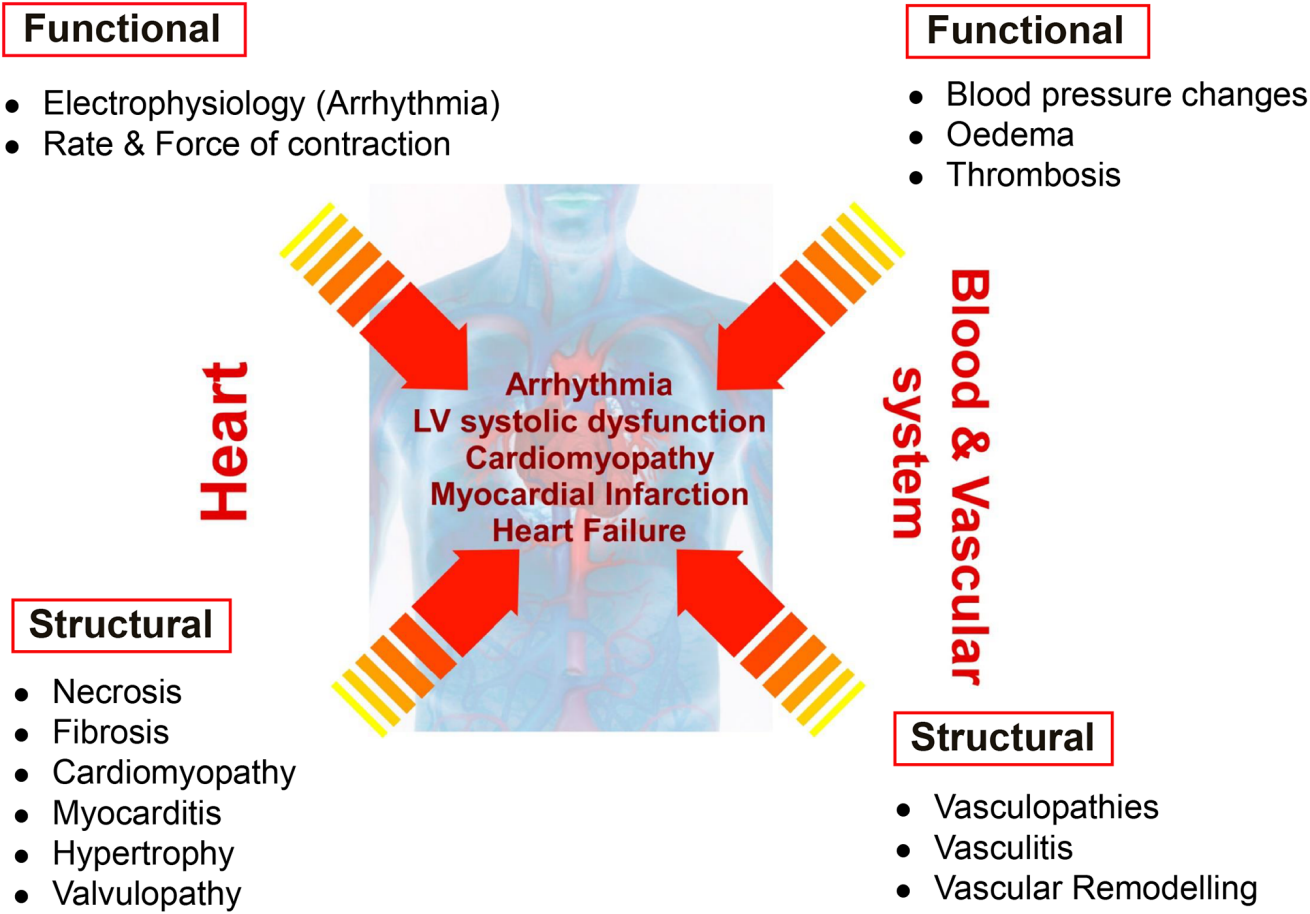
Distinction between functional and structural toxicity. Drug-induced cardiovascular toxicity can potentially affect all components and functions of the cardiovascular system. Functional toxicity can occur in the heart causing effects on cardiomyocyte electrophysiology and rate and force of contraction. Functional toxicity can also occur in the blood and vascular system with adverse effects on blood pressure, oedema and thrombosis. Structural toxicity can cause morphological changes in the heart with effects on cardiomyocytes, valvular endothelial cells, cardiac fibroblasts resulting in a range of pathologies such as necrosis, fibrosis, cardiomyopathy, myocarditis, hypertrophy and valvulopathy. Structural toxicity can also occur in the blood and vascular system with adverse effects on endothelial cells and smooth muscle cells resulting in vasculopathies, vasculitis and vascular remodelling. Structural and functional toxicity within the cardiac and vascular system ultimately clinically manifests in the development of arrhythmia, LV systolic dysfunction, cardiomyopathy, myocardial infarction and heart failure.

**Figure 3:**
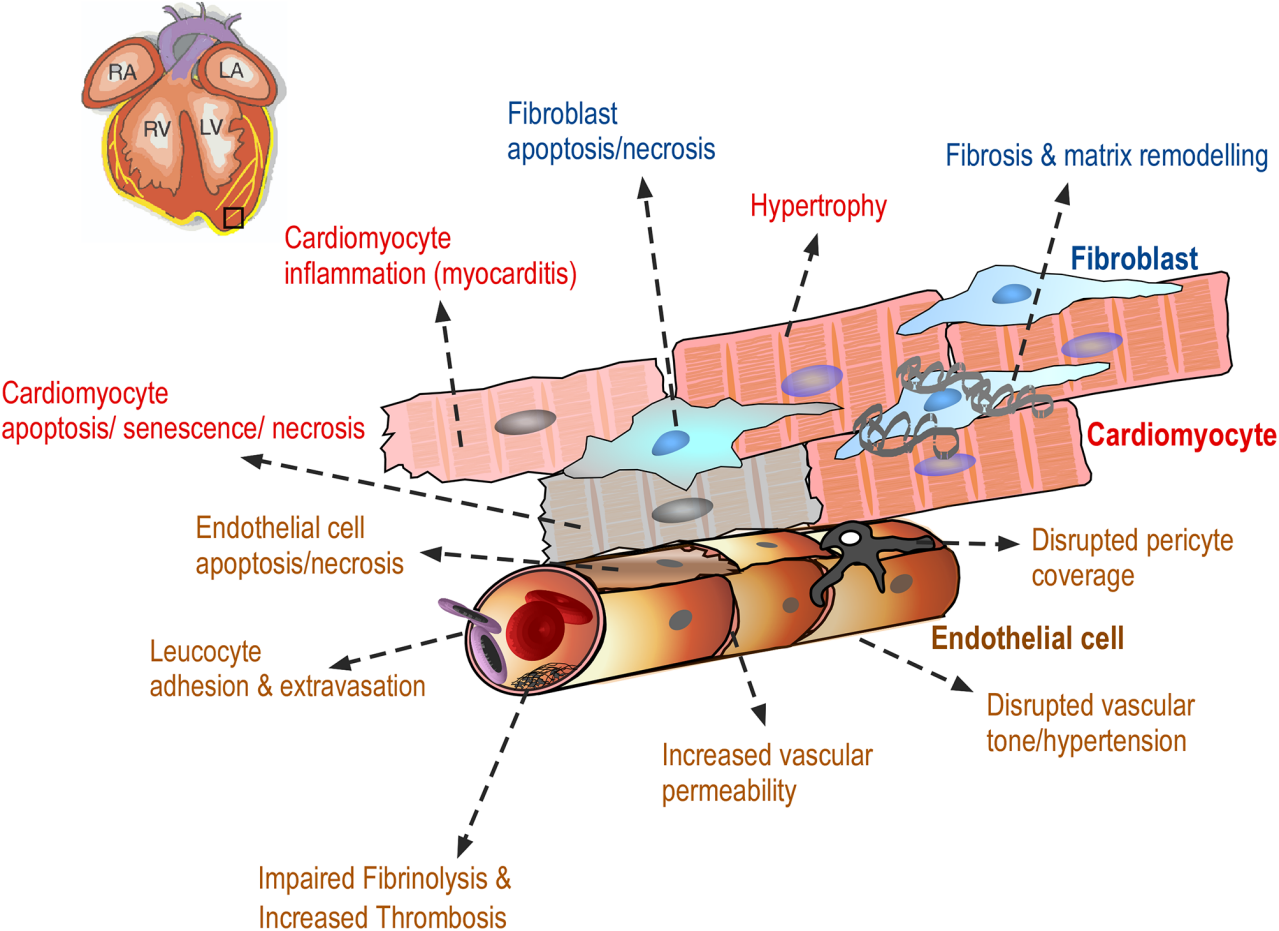
Drug-induced cardiovascular toxicity can affect multiple cells within the myocardium. Diagram showing the adverse effects of anti-cancer drugs on cardiac endothelial cells, vascular pericytes, cardiac fibroblasts and cardiomyocytes resulting in a range of morphological changes in these cells, ultimately contributing to the clinical manifestation of cardiac toxicity. These adverse effects are discussed in detail in the text.

Drug-induced cardiovascular toxicity is defined as a severe and potentially fatal adverse reaction to certain drugs. Cardiotoxicity is a major cause of attrition in preclinical and clinical drug development (40–42). Drug-induced cardiovascular toxicity can affect all components and functions of the cardiovascular system and can be functional, causing alteration of the mechanical function of the heart and vasculature and/or structural, causing morphological changes to cardiomyocytes, endothelial cells, cardiac fibroblasts, ultimately adversely affecting heart and vascular function and culminating in the development of conditions such as arrhythmia, LV systolic dysfunction, cardiomyopathy, myocardial infarction and heart failure (43) (Figure 2). Historically, pre-clinical *in vitro* strategies for the detection of drug-induced cardiovascular toxicity have primarily focused on detecting functional cardiotoxicity with a particular focus on electrophysiology and electrocardiogram (ECG) abnormalities detected by high-throughput screening of key ion channels, for example, hERG, implicated in functional cardiotoxicity (44, 45). However, cardiovascular functional abnormalities can also arise through impaired left ventricular function characterized by changes in cardiomyocyte contractility. Structural cardiotoxicity can occur with a wide range of drugs and is a concern with several classes of anti-cancer agents ranging from classical chemotherapy agents such as anthracyclines, to the more modern molecular targeted therapy aimed at inhibiting specific protein kinases that play a role in cell proliferation in cancer cells (46). These kinases are often expressed in a range of normal cell types in addition to cancer cells, meaning that inadvertent effects in cardiac cells can result in structural changes to specific cardiac cell types over time, which can ultimately render the patient sensitive to the development of cardiac problems including: heart muscle injury with cardiomyopathy and heart failure, complications of coronary artery disease leading to myocardial ischaemia, arrhythmias, hypertension and thromboembolism (47, 48). Whilst doxorubicin remains one of the most widely studied cardiotoxic drugs, adverse drug effects can occur across a range of different anti-cancer drugs (Table 1) and affect multiple cardiac cell types leading to pathological changes that ultimately reduce cardiac function in patients (Figure 3).

**Table 1:** Cardiovascular toxicities associated with anticancer drugs.

Drug Class	Adverse cardiovascular event	Incidence of LVD & HF (%)	Incidence of general ADRs (%)	Reference
Chemotherapy				
**Anthracyclines** Doxorubicin (Adriamycin®)	Arrhythmia, LVD, HF	7–26		(49–51)
**Alkylating agents** Cyclophosphamide (Cytoxan®)	Arrhythmia, LVD, HF,	7–28		(52)
**Anti-metabolites** 5-Flurouracil (Adrucil®)	Coronary vasospasm, Cardiomyopathy & LVD, Arrhythmia	4–12		(53)
**Platinum based therapy** Cisplatin, Carboplatin (Paraplatin®)	Arterial vascular disease, hypertension			(54)
**Microtubule binding drugs** Docetaxel (Taxotere®), Paclitaxel (Taxol®) Vincristine (Oncovin®)	Arrhythmias, LVD, HF	2–8		(52)
Molecular Targeted Therapy				
**Her2 inhibitors** Trastuzumab (Herceptin®) Lapatinib (Tyverb®)	LVD, HF LVD, HF	2–28 1.6		(51) (55)
**VEGF pathway inhibitors** Bevacizumab (Avastin®) Sunitinib (Sutent®)	Hypertension, LVD, HF Hypertension, LVD, HF	2–4 3–8		(56)
**Bcr-Abl inhibitors** Imatinib (Glivec®) Dasatinib (Sprycel®)	LVD, HF Hypertension, Arrhythmia LVD, HF	0.7–1.1 2–4		(51, 57)
**BRaf inhibitors** Vemurafenib (Zelboraf®)	Hypertension, QT prolongation, LVD	9		(58)
**MEK inhibitors** Trametinib (Mekinist®)	Hypertension, LVD	5.2		(59, 60)
Immune Checkpoint Inhibitors (ICIs)				
**CTL4 inhibitors** Ipilimumab (Yervoy®)	General cardiac ADRs: pericarditis, myocarditis, conduction abnormalities		1–1.8	(61)
**PD1 inhibitors** Pembrolizumab (Keytruda®) Nivolumab (Opdivo®)	General cardiac ADRs: myocarditis, pericardial disease, conduction abnormalities		1.99 2.23	(62)
**PDL1 inhibitors** Atezolizumab (Tecentriq^®^)	General cardiac ADRs: myocarditis, pericardial disease, conduction abnormalities		2.59	(62)

ADRs, adverse drug reactions; HF, heart failure, LVD, left ventricular dysfunction.

### 3.1 Adverse effects on endothelial cells

Endothelial cells line the blood vessels of the coronary microcirculation, arteries and veins regulating the permeability and tone (63). Furthermore, endothelial cells also regulate immune cell adhesion and suppress thrombosis. Endothelial cells are in intimate contact with the blood and xenobiotics, and can be adversely affected by cancer drugs in a number of ways (Figure 3).

#### 3.1.1 Increased vascular permeability

Vascular toxicity in response to chemotherapy often reflects endothelial dysfunction with loss of vasorelaxant effects and suppressed anti-inflammatory and vascular reparative functions (64). These effects can exacerbate underlying conditions of hypertension, thrombosis and atherosclerosis. Furthermore, chemotherapy can also promote coagulation of platelets due to reduced nitric oxide bioavailability (65). Vascular permeability regulates the movement of solutes, xenobiotics and immune cells from the circulation to the underlying tissues and is critical for normal vascular homeostasis. Vascular permeability is regulated by endothelial tight junctions and adherens junctions (66). *In vivo* studies in a rat model of doxorubicin injury have shown increased cardiac capillary permeability following doxorubicin treatment (67). *In vitro* studies using bovine pulmonary artery endothelial cells have shown that doxorubicin can increase endothelial permeability (68). Recent *in vitro* data have shown that the anti-cancer drugs doxorubicin and trastuzumab (Herceptin) can adversely affect human cardiac microvascular endothelial cell barrier function by reducing expression of the tight junction protein ZO-1, leading to increased drug permeability (69, 70).

#### 3.1.2 Apoptosis

Apoptosis is a form of programmed cell death that occurs in multicellular organisms (71). Whilst the exact mechanism of doxorubicin induced cardiotoxicity is still unknown, studies in rats have shown that doxorubicin is able to induce both cardiomyocyte and endothelial cell apoptosis (72). Organ culture of rabbit mesenteric arteries treated with doxorubicin revealed endothelial apoptosis and impairment of endothelium dependent relaxation (73). Recent data from a mouse model of doxorubicin-induced cardiotoxicity has shown that doxorubicin-induced endothelial cell apoptosis and cardiotoxicity are alleviated by treatment with VEGF-B, which acts primarily on its cognate receptor, VEGFR-1, expressed on vascular endothelial cells (74). This novel study shows that specifically targeting the endothelium could be a potential therapeutic strategy to protect against doxorubicin induced cardiotoxicity. Clinical data from children receiving doxorubicin, in addition to other chemotherapy, has shown reduced brachial artery vasomotor reactivity months after treatment ended (75). Primary endothelial damage is the mechanism of cardiotoxicity of tubulin-binding drugs, such as vinblastine and vincristine, which have been shown to increase mitotic arrest and endothelial cell apoptosis (76).

#### 3.1.3 Dysregulated angiogenesis

Angiogenesis is the formation of new blood vessels from pre-existing vasculature and is important for efficient cardiac vascularization (77). *In vitro* experiments using rat cardiac microvascular endothelial cells have shown that doxorubicin stabilises hypoxia inducible factor 1-α (HIF-1α) levels resulting in the increased transcription of *VEGF* mRNA and release of VEGF-A and down-regulation of VEGFR-2 levels (78). Recent data has also shown that doxorubicin is also able to down-regulate VEGFR-2 levels and cause cellular senescence in endothelial cells (79).

#### 3.1.4 Disrupted vascular tone/hypertension

Vascular tone refers to the degree of constriction experienced by a blood vessel relative to its maximally dilated state. All arterial and venous vessels under basal conditions exhibit some degree of smooth muscle contraction that determines the diameter, and hence tone, of the vessel. Vascular tone is determined by many different competing vasoconstrictor and vasodilator influences acting on the blood vessel. These influences can be separated into *extrinsic* factors such as the sympathetic and parasympathetic nervous system, and *intrinsic factors* that originate from the vessel itself or the surrounding tissue such as adenosine and nitric oxide (NO) (80, 81). NO is a natural vasodilator, acting on smooth muscle cells to promote vasodilation. Angiogenesis inhibitors targeting the vascular endothelial growth factor (VEGF) signaling pathway have been important additions in the therapy of various cancers, especially renal cell carcinoma and colorectal cancer. Bevacizumab (Avastin) targets the VEGF-A ligand whereas TKis such as sunitinib, sorafenib, pazopanib and regorafenib target VEGFR-2 and other RTKs. All of these anti-angiogenic drugs lead to hypertension, which is thought to occur by loss of VEGF-mediated NO synthase activity, reducing the level of NO production in the walls of arterioles, ultimately increasing vascular tone (56). A marked increase in systemic hypertension results in significant increases in the afterload of left ventricle and myocardial oxygen demand leading to myocardial injury/infarction and LV systolic dysfunction in vulnerable patients. The incidence of hypertension has been estimated at 15%–47% with sunitinib and 17%–42% in patients treated with sorafenib (82).

Cyclophosphamide is thought to suppress NO production via a different mechanism to anti-angiogenic drugs, by generating free radicals which react with NO to produce peroxynitrite (ONOO−), thus reducing the level of free NO (83). Vasospasm refers to an acute condition in which an arterial spasm leads to vasoconstriction. Coronary artery vasospasm can lead to reduced blood flow and ischaemia. 5-FU can worsen or aggravate vascular spasm, resulting in acute coronary syndrome, peripheral arterial disease, cerebrovascular disease, or stress-induced cardiomyopathy (84).

#### 3.1.5 Leukocyte adhesion and transmigration

Leukocyte migration into the vessel wall is an early pathological event in the progression of a number of cardiovascular pathologies such as atherosclerosis, diabetic cardiomyopathy and heart failure (85, 86). In response to cytokines and pro-inflammatory mediators, the endothelial lining of the microvasculature will increase expression of intracellular adhesion molecule 1 (ICAM-1) and/or vascular cell adhesion molecule (VCAM-1) that interact with leukocyte-expressed integrins allowing adhesion to the luminal surface of endothelial cells and transmigration through endothelial junctions to the underlying myocardium. Immune checkpoint inhibitors (ICIs) are a recently developed class of anti-cancer drugs, consisting of humanised monoclonal antibodies that target T lymphocytes to prevent down-regulation of the immune response. Iplimumab (Yervoy®) targets CTLA4, nivolumab (Opdivo®) and pembrolizumab (Keytruda) target PD-1, whilst atezolizumab (Tecentriq®) targets PDL-1. There is a growing awareness that these molecules can cause adverse cardiac effects such as atrial fibrillation, ventricular arrhythmia and most seriously, inflammation of the myocardium, termed myocarditis, occurring in up to 1% of patients taking immune checkpoint inhibitors (87, 88). The exact mechanism of myocarditis is not fully understood, however, as in viral myocarditis, patients with ICI-related myocarditis have been shown to have T-cell infiltration of the myocardium consistent with inflammation (89).

#### 3.1.6 Impaired fibrinolysis/increased thrombosis

Haemostasis is a complex biochemical response to injury allowing the formation of a blood clot and repair of damaged endothelium. The maintenance of the equilibrium between coagulation and fibrinolysis is vital, as imbalance can lead to abnormal bleeding or increased risk of thrombosis (90). VEGF inhibitors (bevacizumab, sunitinib, sorafenib) can cause endothelial dysfunction and platelet function inhibition, resulting in arterial thrombosis in approximately 4% of patients in addition to venous thrombosis. Alkylating agents such as cisplatin can trigger platelet aggregation (91). Recent data has shown that cyclophosphamide can potentially induce a prothrombotic state in endothelial cells by upregulating the release of prostacyclin (PGI_2_) and thromboxane A2 (TXA_2_) (92).

#### 3.1.7 Disrupted pericyte coverage

Pericytes are mural cells which stabilise capillary blood vessels through direct contact with endothelial cells on the abluminal surface of the blood vessel (27). The absence of pericytes can lead to vascular leakage and haemorrhaging (93). Sunitinib, a TKi used to treat metastatic renal cancer, has been shown to deplete cardiac microvascular pericytes resulting in changes in the cardiac microvasculature coverage in a mouse study of cardiotoxicity (94). Interestingly, coverage of skeletal muscle pericytes was not affected by sunitinib. This effect of sunitinib on cardiac pericytes was via inhibition of PDGFR-β signalling and was not reproduced by doxorubicin.

### 3.2 Adverse effects on cardiomyocytes

Cardiac muscle contraction is a strictly regulated process which coordinates a series of electrophysiological, biochemical and mechanical events, resulting in the ejection of blood from the right ventricle into the pulmonary circulation and left ventricle into the systemic circulation. LV dysfunction induced by cardiotoxic chemotherapies is defined by a decrease in left ventricular ejection fraction (LVEF) of >10 percentage points to a value <53% and is usually monitored by echocardiography (95). To detect early myocardial damage before a change in LVEF is evident, levels of biomarkers such as cardiac troponin-I are monitored to detect any increase in plasma concentration. Cardiac troponin I (cTnI) and cardiac troponin T (cTnT) are cardiomyocyte specific proteins that form the troponin complex with thick filaments in the contractile sarcomeres of cardiomyocytes. Both cTnI and cTnT are released into the bloodstream on acute myocardial injury, possibly due to necrosis of cardiomyocytes (96). Human adult cardiomyocytes have a restricted replicative and regenerative capacity (18, 97, 98), so irreversible damage from xenobiotics can have an accumulative effect over time in reducing cardiac contractile function. Damage to the myocardial cells can occur due to reduced blood supply in ischaemia or via direct drug-induced toxicity, leading to changes in myocardial morphology, physiology and biochemistry.

#### 3.2.1 Apoptosis

Cardiomyocyte cell death is thought to be regulated by a number of mechanisms such as apoptosis and necrosis. Exposure to doxorubicin is known to lead to cardiomyocyte cell death via a number of different mechanisms: generation of reactive oxygen species (ROS) leading to damage to lipids, DNA and proteins and topoisomerase 2 beta (TOP2B) inhibition leading to DNA strand breaks in cardiomyocytes and mitochondrial damage (99). Analysis of doxorubicin's effects in studies using rat cardiomyocytes and *in vivo* analysis has shown caspase 3 activation (100) and cytochrome c release (101). *In vitro* analysis using human pluripotent stem cell-derived cardiomyocytes (hiPS-CMs) has shown that doxorubicin induces apoptosis through upregulation of death receptors (102). Other forms of regulated cell death such autophagy, ferroptosis and necroptosis have also been implicated in doxorubicin injury (103), suggesting that doxorubicin has the potential to activate multiple pathways leading to cardiomyocyte death.

Classical chemotherapy drugs have also been shown to induce apoptosis in cardiomyocytes. Cisplatin and cyclophosphamide can induce ROS and mitochondrial dysfunction in rat cardiomyocytes (104, 105).

Trastuzumab (Herceptin) binds to the HER2 protein, which is upregulated on certain breast cancer cells, but can also bind to HER2 expressed on cardiomyocytes, interfering with the normal NRG-1/ErbB2 signalling axis resulting in cardiomyocyte apoptosis (106). Trastuzumab (Herceptin) induces cardiomyocyte toxicity through a mitochondrial pathway depending on ROS production and oxidative stress and is reversed by the antioxidant N-acetyl cysteine (NAC) (107). HER2 inhibition by trastuzumab is associated with an increase in expression of the proapoptotic Bcl-xS and decreased levels of antiapoptotic Bcl-xL. These alterations induce mitochondrial dysfunctions such as loss of mitochondrial membrane potential (ΔΨ), and ATP depletion with the disruption of cardiomyocyte cellular energetic and reversible contractile impairment (108). Trastuzumab-mediated blockade of ErbB2 signalling increases anthracycline-induced cardiotoxicity, most probably due to the suppression of the protective NRG-1/ErbB2 signalling axis resulting in the augmentation of anthracycline toxicity (109).

Molecular targeting drugs inhibit RTKs present on cardiomyocytes affecting cell survival. Sunitinib, which inhibits a broad range of RTKs such as PDGFR, VEGFR, c-Kit, has been shown to adversely affect mitochondrial function and cause cardiomyocyte apoptosis in animal studies (110). Furthermore, studies utilizing endomyocardial biopsies from two patients with gastrointestinal stromal tumours (GIST), who had developed severe left ventricular dysfunction during sunitinib treatment, revealed mitochondrial structure abnormalities following TEM analysis of cardiac sections (82).

#### 3.2.2 Hypertrophy

Cardiac hypertrophy is an abnormal enlargement or thickening of the heart muscle resulting from an increase in the size of cardiomyocytes. It is an adaptive response to pressure or volume stress as a result of hypertension or valvular disease, and can also occur from mutations of sarcomeric proteins, or loss of contractile mass from prior infarction (111). Anti-cancer drugs have the potential to indirectly cause cardiac hypertrophy by reducing cardiac performance. Sunitinib has been reported to induce cardiac hypertrophy in mice by inducing cardiac remodeling and increasing cardiac glycolytic metabolism resulting in fibrosis, increased LV mass and increased EF (112).

#### 3.2.3 Myocarditis and immune cell infiltration

Myocarditis is an inflammatory disease of the myocardium that can be caused by a viral or bacterial infection or an auto-immune disease (113). The advent of immune checkpoint inhibitor (ICI) therapy has heralded a new era in cancer therapy with drugs targeting CTLA4 (ipilimumab) and PD-1 (nivolumab, pembrolizumab) on activated T-cells, and PD-L1 (atezolizumab) on epithelial and antigen presenting cells (APC). However, ICI therapy is also associated with a spectrum of immune-related adverse events (irAEs) such as colitis, hepatitis and myocarditis. ICI-associated myocarditis occurs in approximately 1% of patients and can be severe and life threatening (89). It is characterized by CD4 + ve T-cell and macrophage infiltration into the myocardium, ultimately resulting in myocyte damage and cardiac dysfunction. Previous genetic knockout studies in mice have shown that loss of immune checkpoint protein function can cause cardiac problems; deficiency of CTLA-4 in mice is associated with a severe autoimmune myocarditis (114) whilst PD-1 or PD-L1 deficient mice are susceptible to autoimmune myocarditis (115–117). Immune cell infiltration is thought to contribute to cardiotoxicity observed with other anti-cancer drugs. Studies in mouse models of doxorubicin-induced acute cardiotoxicity have shown a significant infiltration of neutrophils into hearts 24 h after doxorubicin treatment, which was accompanied by an acute and late decrease in cardiac function, disruption of the vascular endothelium, and an increase in collagen deposition, leading to fibrosis. The depletion of neutrophils prevented doxorubicin-induced cardiotoxicity with the preservation of vascular structures and prevention of excess collagen deposition (118, 119). Another mouse study has shown that doxorubicin treatment enhanced the pro-inflammatory M1 macrophage derived from monocytes and suppressed the reparative M2 macrophage population present within the heart (120).

#### 3.2.4 Senescence

Cellular senescence is a phenomenon characterized by stable cell cycle arrest, mitochondrial dysfunction and cessation of cell division (121). Senescence is a hallmark of aging and accumulation of senescent cells over time is associated with the declining function of the aged cardiovascular system and many age-related CVDs, including atherosclerosis and heart failure (122). *In vivo* studies in rodents have shown that doxorubicin can induce biomarkers of senescence in the myocardium of mice (123) and rats (124). Analysis of LV human heart tissue from patients with doxorubicin-induced cardiotoxicity has revealed increased expression of a range of markers of senescence in cardiomyocytes compared to LV tissue from healthy donors (125). In addition to cardiomyocytes, doxorubicin-induced senescence can potentially occur in cardiac fibroblasts and cardiac endothelial cells (126).

### 3.3 Adverse effects on cardiac fibroblasts

In response to cardiac injury, both cytokine and neurohumoral factors are released and profound changes occur in the mechanical strain relationships within the ventricular wall (or septum), which together are thought to underlie fibroblast activation. In response to various stressors, cardiac fibroblasts trans-differentiate into myofibroblasts, which synthesize larger amounts of ECM and gain contractile activity due to expression of *α*-smooth muscle actin (α-SMA) (127).

#### 3.3.1 Apoptosis and necrosis

The potential for cardiac fibroblasts to undergo apoptosis in response to anti-cancer drugs has not been widely studied. However, a recent study has shown that doxorubicin caused both apoptosis of cardiac fibroblasts and secretion of Fas ligand, which in turn promoted cardiomyocyte death in a paracrine manner (128). Furthermore, conditional deletion of ataxia telangiectasia mutated kinase (ATM) in mouse cardiac fibroblasts attenuated cardiac cell apoptosis, Left Ventricular Dysfunction (LVD), and mortality in response to doxorubicin, suggesting that fibroblasts are central in the pathogenesis of doxorubicin cardiotoxicity through ATM.

#### 3.3.2 Cardiac fibrosis and matrix remodelling

Accumulation of fibrotic tissue is one of the underlying features of doxorubicin-induced cardiomyopathy triggered by inflammation and free radical ROS generation. Doxorubicin has been shown to induce ROS and TGF-β and transformation of cardiac fibroblasts to myofibroblasts in a rat model of cardiotoxicity (129). The myofibroblasts express contractile proteins such as α-smooth muscle actin (*α*-SMA) and secrete pro-fibrotic factors such as collagen type 1 and fibronectin. Another study in rats has shown that doxorubicin acts on cardiac fibroblasts, independent of cardiomyocyte injury, to produce excess collagen (130). *In vitro* studies have also shown that doxorubicin induces α-SMA and trans-differentiation in human cardiac fibroblasts. Furthermore, induction of matrix metalloprotease-I (MMP-1) and IL-6 was also reported indicating the potential for matrix remodelling and inflammatory cell recruitment (131). Together, this data suggests that adverse effects of doxorubicin on cardiac fibroblasts can result in long-term changes in matrix remodelling, potentially disrupting the stromal environment around cardiomyocytes leading to long-term changes in cardiac physiology.

## 4 Conclusions and future perspectives

Cancer therapies have evolved remarkably from classical chemotherapy, first developed in the 1950s–1970s to target rapidly dividing cells, through molecular targeted therapies developed in the 1990s to target specific signalling pathways and more recently to immunotherapies harnessing the potential of the immune system to target cancer cells. As patients with cancer are living longer, the potential for drug-induced cardiovascular toxicity is also increasing. Furthermore, patients may be treated with a combination of therapies which can increase therapeutic efficacy with a concomitant increase in toxicity. Research over the last two decades has started to reveal the complexity of cardiovascular toxicity induced by anti-cancer drugs and highlighted the potential to adversely affect multiple cardiac cell types. This has profound implications for current and future approaches to pre-clinical cardiovascular safety assessment (132). Historically, drug safety assessment has focused on analysing electrophysiological effects in cardiomyocytes, or ion channel expressing cell lines, with pathological assessment of organ toxicity in rodents and dogs (133, 134). There is a clear need for more advanced *in vitro* cardiac cell models that mirror human cardiac physiology.

The advent of iPSC-derived human cardiomyocytes has allowed the development of contractile-competent 3D cardiac microtissues that combine cardiomyocytes, endothelial cells and fibroblasts (135–139). These multi-cellular cardiac cell models are amenable to plate based high content imaging and other technologies to assess effects on contractility and cell morphology (140, 141), allowing simultaneous assessment of both functional and structural cardiotoxicity.

Progression in the development of suitable cardiac endothelial and fibroblast iPSC differentiation protocols, alongside iPSC derived cardiomyocytes containing disease relevant mutations, will enable the formation of cardiac microtissue derived entirely from iPSC sources. Advantages include provision of cells at scale suitable for high volume toxicity profiling in wild type and disease models, and the potential to derive cells from multiple gender and ethnic backgrounds (20). This may yield cardiac models that better translate to patients reflecting clinically observed differences in cardiotoxicity typically revealed only during later stage clinical trials or post marketing.

Going forward, the challenges with *in vitro* cardiac cell models are to try and recapitulate haemodynamic flow, where endothelial cells form lumen-containing vessels surrounded by cardiac pericytes and stromal cardiac fibroblasts and cardiomyocytes. These models will allow us to recapitulate the physiological environment in which xenobiotics are delivered to the heart and allow more physiologically relevant analysis of drug-induced changes in cardiac physiology, this level of complexity is a real challenge for heart-on-a-chip approaches. As our understanding of the multi-cellular nature of cancer drug induced cardiotoxicity increases, so does the potential to exploit this knowledge to identify new biomarkers of cardiac cell injury as well as therapeutic interventions to try and ameliorate the adverse effects of drugs on cardiac cells.

## References

[1] Herrmann J. Adverse cardiac effects of cancer therapies: cardiotoxicity and arrhythmia. Nat Rev Cardiol. (2020) 17(8):474–502. 10.1038/s41569-020-0348-1 32231332 PMC8782611

[2] Kostakou PM Kouris NT Kostopoulos VS Damaskos DS Olympios CD. Cardio-oncology: a new and developing sector of research and therapy in the field of cardiology. Heart Fail Rev. (2019) 24(1):91–100. 10.1007/s10741-018-9731-y 30073443

[3] Koutsoukis A Ntalianis A Repasos E Kastritis E Dimopoulos MA Paraskevaidis I. Cardio-oncology: a focus on cardiotoxicity. Eur Cardiol. (2018) 13(1):64–9. 10.15420/ecr.2017:17:2 30310475 PMC6159462

[4] Minami M Matsumoto S Horiuchi H. Cardiovascular side-effects of modern cancer therapy. Circ J. (2010) 74(9):1779–86. 10.1253/circj.cj-10-0632 20716834

[5] Morelli MB Bongiovanni C Da Pra S Miano C Sacchi F Lauriola M Cardiotoxicity of anticancer drugs: molecular mechanisms and strategies for cardioprotection. Front Cardiovasc Med. (2022) 9:847012. 10.3389/fcvm.2022.847012 35497981 PMC9051244

[6] Xie S Yang Y Luo Z Li X Liu J Zhang B Role of non-cardiomyocytes in anticancer drug-induced cardiotoxicity: a systematic review. iScience. (2022) 25(11):105283. 10.1016/j.isci.2022.105283 36300001 PMC9589207

[7] Rossant J Howard L. Signaling pathways in vascular development. Annu Rev Cell Dev Biol. (2002) 18:541–73. 10.1146/annurev.cellbio.18.012502.105825 12142271

[8] Aguilar-Sanchez C Michael M Pennings S. Cardiac stem cells in the postnatal heart: lessons from development. Stem Cells Int. (2018) 2018:1247857. 10.1155/2018/1247857 30034478 PMC6035836

[9] Vegh AMD Duim SN Smits AM Poelmann RE Ten Harkel ADJ DeRuiter MC Part and parcel of the cardiac autonomic nerve system: unravelling its cellular building blocks during development. J Cardiovasc Dev Dis. (2016) 3(3):28. 10.3390/jcdd3030028 29367572 PMC5715672

[10] Laugwitz KL Moretti A Caron L Nakano A Chien KR. Islet1 cardiovascular progenitors: a single source for heart lineages? Development. (2008) 135(2):193–205. 10.1242/dev.001883 18156162

[11] Garry DJ Olson EN. A common progenitor at the heart of development. Cell. (2006) 127(6):1101–4. 10.1016/j.cell.2006.11.031 17174889

[12] Gittenberger-de Groot AC Bartelings MM Deruiter MC Poelmann RE. Basics of cardiac development for the understanding of congenital heart malformations. Pediatr Res. (2005) 57(2):169–76. 10.1203/01.PDR.0000148710.69159.61 15611355

[13] Carmona R Barrena S Lopez Gambero AJ Rojas A Munoz-Chapuli R. Epicardial cell lineages and the origin of the coronary endothelium. FASEB J. (2020) 34(4):5223–39. 10.1096/fj.201902249RR 32068311

[14] Brutsaert DL. Cardiac endothelial-myocardial signaling: its role in cardiac growth, contractile performance, and rhythmicity. Physiol Rev. (2003) 83(1):59–115. 10.1152/physrev.00017.2002 12506127

[15] Lother A Kohl P. The heterocellular heart: identities, interactions, and implications for cardiology. Basic Res Cardiol. (2023) 118(1):30. 10.1007/s00395-023-01000-6 37495826 PMC10371928

[16] Yuan X Braun T. Multimodal regulation of cardiac myocyte proliferation. Circ Res. (2017) 121(3):293–309. 10.1161/CIRCRESAHA.117.308428 28729454

[17] van Berlo JH Molkentin JD. An emerging consensus on cardiac regeneration. Nat Med. (2014) 20(12):1386–93. 10.1038/nm.3764 25473919 PMC4418535

[18] Bergmann O Zdunek S Felker A Salehpour M Alkass K Bernard S Dynamics of cell generation and turnover in the human heart. Cell. (2015) 161(7):1566–75. 10.1016/j.cell.2015.05.026 26073943

[19] Li F Wang X Capasso JM Gerdes AM. Rapid transition of cardiac myocytes from hyperplasia to hypertrophy during postnatal development. J Mol Cell Cardiol. (1996) 28(8):1737–46. 10.1006/jmcc.1996.0163 8877783

[20] Pang L. Toxicity testing in the era of induced pluripotent stem cells: a perspective regarding the use of patient-specific induced pluripotent stem cell-derived cardiomyocytes for cardiac safety evaluation. Curr Opin Toxicol. (2020) 23-24:50–5. 10.1016/j.cotox.2020.04.001

[21] Patterson M Barske L Van Handel B Rau CD Gan P Sharma A Frequency of mononuclear diploid cardiomyocytes underlies natural variation in heart regeneration. Nat Genet. (2017) 49(9):1346–53. 10.1038/ng.3929 28783163 PMC5736145

[22] Gunthel M Barnett P Christoffels VM. Development, proliferation, and growth of the mammalian heart. Mol Ther. (2018) 26(7):1599–609. 10.1016/j.ymthe.2018.05.022 29929790 PMC6037201

[23] Pinto AR Ilinykh A Ivey MJ Kuwabara JT D'Antoni ML Debuque R Revisiting cardiac cellular composition. Circ Res. (2016) 118(3):400–9. 10.1161/CIRCRESAHA.115.307778 26635390 PMC4744092

[24] Colliva A Braga L Giacca M Zacchigna S. Endothelial cell-cardiomyocyte crosstalk in heart development and disease. J Physiol. (2020) 598(14):2923–39. 10.1113/JP276758 30816576 PMC7496632

[25] Parodi EM Kuhn B. Signalling between microvascular endothelium and cardiomyocytes through neuregulin. Cardiovasc Res. (2014) 102(2):194–204. 10.1093/cvr/cvu021 24477642 PMC3989448

[26] Lemmens K Doggen K De Keulenaer GW. Role of neuregulin-1/Erbb signaling in cardiovascular physiology and disease: implications for therapy of heart failure. Circulation. (2007) 116(8):954–60. 10.1161/CIRCULATIONAHA.107.690487 17709650

[27] Nees S Weiss DR Juchem G. Focus on cardiac pericytes. Pflugers Arch. (2013) 465(6):779–87. 10.1007/s00424-013-1240-1 23443852

[28] Avolio E Campagnolo P Katare R Madeddu P. The role of cardiac pericytes in health and disease: therapeutic targets for myocardial infarction. Nat Rev Cardiol. (2024) 21(2):106–18. 10.1038/s41569-023-00913-y 37542118

[29] Moore-Morris T Guimaraes-Camboa N Banerjee I Zambon AC Kisseleva T Velayoudon A Resident fibroblast lineages mediate pressure overload-induced cardiac fibrosis. J Clin Invest. (2014) 124(7):2921–34. 10.1172/JCI74783 24937432 PMC4071409

[30] Mikawa T Gourdie RG. Pericardial mesoderm generates a population of coronary smooth muscle cells migrating into the heart along with ingrowth of the epicardial organ. Dev Biol. (1996) 174(2):221–32. 10.1006/dbio.1996.0068 8631495

[31] Wessels A van den Hoff MJ Adamo RF Phelps AL Lockhart MM Sauls K Epicardially derived fibroblasts preferentially contribute to the parietal leaflets of the atrioventricular valves in the murine heart. Dev Biol. (2012) 366(2):111–24. 10.1016/j.ydbio.2012.04.020 22546693 PMC3358438

[32] Ali SR Ranjbarvaziri S Talkhabi M Zhao P Subat A Hojjat A Developmental heterogeneity of cardiac fibroblasts does not predict pathological proliferation and activation. Circ Res. (2014) 115(7):625–35. 10.1161/CIRCRESAHA.115.303794 25037571

[33] Baudino TA Carver W Giles W Borg TK. Cardiac fibroblasts: friend or foe? Am J Physiol Heart Circ Physiol. (2006) 291(3):H1015–26. 10.1152/ajpheart.00023.2006 16617141

[34] Pellman J Zhang J Sheikh F. Myocyte-fibroblast communication in cardiac fibrosis and arrhythmias: mechanisms and model systems. J Mol Cell Cardiol. (2016) 94:22–31. 10.1016/j.yjmcc.2016.03.005 26996756 PMC4861678

[35] Kohl P Camelliti P Burton FL Smith GL. Electrical coupling of fibroblasts and myocytes: relevance for cardiac propagation. J Electrocardiol. (2005) 38(4 Suppl):45–50. 10.1016/j.jelectrocard.2005.06.096 16226073

[36] Fan D Takawale A Lee J Kassiri Z. Cardiac fibroblasts, fibrosis and extracellular matrix remodeling in heart disease. Fibrogenesis Tissue Repair. (2012) 5(1):15. 10.1186/1755-1536-5-15 22943504 PMC3464725

[37] Ma Y de Castro Bras LE Toba H Iyer RP Hall ME Winniford MD Myofibroblasts and the extracellular matrix network in post-myocardial infarction cardiac remodeling. Pflugers Arch. (2014) 466(6):1113–27. 10.1007/s00424-014-1463-9 24519465 PMC4033805

[38] Souders CA Bowers SL Baudino TA. Cardiac fibroblast: the renaissance cell. Circ Res. (2009) 105(12):1164–76. 10.1161/CIRCRESAHA.109.209809 19959782 PMC3345531

[39] Kamkin A Kiseleva I Lozinsky I Scholz H. Electrical interaction of mechanosensitive fibroblasts and myocytes in the heart. Basic Res Cardiol. (2005) 100(4):337–45. 10.1007/s00395-005-0529-4 15822004

[40] Abassi YA Xi B Li N Ouyang W Seiler A Watzele M Dynamic monitoring of beating periodicity of stem cell-derived cardiomyocytes as a predictive tool for preclinical safety assessment. Br J Pharmacol. (2012) 165(5):1424–41. 10.1111/j.1476-5381.2011.01623.x 21838757 PMC3372727

[41] Albini A Pennesi G Donatelli F Cammarota R De Flora S Noonan DM. Cardiotoxicity of anticancer drugs: the need for cardio-oncology and cardio-oncological prevention. J Natl Cancer Inst. (2010) 102(1):14–25. 10.1093/jnci/djp440 20007921 PMC2802286

[42] Laverty H Benson C Cartwright E Cross M Garland C Hammond T How can we improve our understanding of cardiovascular safety liabilities to develop safer medicines? Br J Pharmacol. (2011) 163(4):675–93. 10.1111/j.1476-5381.2011.01255.x 21306581 PMC3111672

[43] Cross MJ Berridge BR Clements PJ Cove-Smith L Force TL Hoffmann P Physiological, pharmacological and toxicological considerations of drug-induced structural cardiac injury. Br J Pharmacol. (2015) 172(4):957–74. 10.1111/bph.12979 25302413 PMC4314188

[44] Pollard CE Valentin JP Hammond TG. Strategies to reduce the risk of drug-induced Qt interval prolongation: a pharmaceutical company perspective. Br J Pharmacol. (2008) 154(7):1538–43. 10.1038/bjp.2008.203 18500356 PMC2492107

[45] Priest BT Bell IM Garcia ML. Role of herg potassium channel assays in drug development. Channels (Austin). (2008) 2(2):87–93. 10.4161/chan.2.2.6004 18849661

[46] Wang Y. Mitogen-activated protein kinases in heart development and diseases. Circulation. (2007) 116(12):1413–23. 10.1161/CIRCULATIONAHA.106.679589 17875982 PMC3808829

[47] Stortecky S Suter TM. Insights into cardiovascular side-effects of modern anticancer therapeutics. Curr Opin Oncol. (2010) 22(4):312–7. 10.1097/CCO.0b013e32833ab6f1 20535072

[48] Heineke J Molkentin JD. Regulation of cardiac hypertrophy by intracellular signalling pathways. Nat Rev Mol Cell Biol. (2006) 7(8):589–600. 10.1038/nrm1983 16936699

[49] Swain SM Whaley FS Ewer MS. Congestive heart failure in patients treated with doxorubicin: a retrospective analysis of three trials. Cancer. (2003) 97(11):2869–79. 10.1002/cncr.11407 12767102

[50] McGowan JV Chung R Maulik A Piotrowska I Walker JM Yellon DM. Anthracycline chemotherapy and cardiotoxicity. Cardiovasc Drugs Ther. (2017) 31(1):63–75. 10.1007/s10557-016-6711-0 28185035 PMC5346598

[51] Yeh ET Bickford CL. Cardiovascular complications of cancer therapy: incidence, pathogenesis, diagnosis, and management. J Am Coll Cardiol. (2009) 53(24):2231–47. 10.1016/j.jacc.2009.02.050 19520246

[52] Pizzino F Vizzari G Bomzer CA Qamar R Carerj S Zito C Diagnosis of chemotherapy-induced cardiotoxicity. J Patient Centered Res Rev. (2014) 1(3):121–7. 10.17294/2330-0698.1025

[53] Yuan C Parekh H Allegra C George TJ Starr JS. 5-Fu induced cardiotoxicity: case series and review of the literature. Cardiooncology. (2019) 5:13. 10.1186/s40959-019-0048-3 32154019 PMC7048125

[54] Haugnes HS Wethal T Aass N Dahl O Klepp O Langberg CW Cardiovascular risk factors and morbidity in long-term survivors of testicular cancer: a 20-year follow-up study. J Clin Oncol. (2010) 28(30):4649–57. 10.1200/JCO.2010.29.9362 20855830

[55] Moy B Goss PE. Lapatinib-associated toxicity and practical management recommendations. Oncologist. (2007) 12(7):756–65. 10.1634/theoncologist.12-7-756 17673607

[56] Touyz RM Herrmann J. Cardiotoxicity with vascular endothelial growth factor inhibitor therapy. NPJ Precis Oncol. (2018) 2:13. 10.1038/s41698-018-0056-z 30202791 PMC5988734

[57] Nodzon L Fadol A Tinsley S. Cardiovascular adverse events and mitigation strategies for chronic myeloid leukemia patients receiving tyrosine kinase inhibitor therapy. J Adv Pract Oncol. (2022) 13(2):127–42. 10.6004/jadpro.2022.13.2.4 35369400 PMC8955565

[58] Bronte E Bronte G Novo G Bronte F Bavetta MG Lo Re G What links braf to the heart function? New insights from the cardiotoxicity of Braf inhibitors in cancer treatment. Oncotarget. (2015) 6(34):35589–601. 10.18632/oncotarget.5853 26431495 PMC4742127

[59] Flaherty KT Robert C Hersey P Nathan P Garbe C Milhem M Improved survival with Mek inhibition in Braf-mutated melanoma. N Engl J Med. (2012) 367(2):107–14. 10.1056/NEJMoa1203421 22663011

[60] Abdel-Rahman O ElHalawani H Ahmed H. Risk of selected cardiovascular toxicities in patients with cancer treated with Mek inhibitors: a comparative systematic review and meta-analysis. J Glob Oncol. (2015) 1(2):73–82. 10.1200/JGO.2015.000802 28804776 PMC5539872

[61] Weber J Thompson JA Hamid O Minor D Amin A Ron I A randomized, double-blind, placebo-controlled, phase II study comparing the tolerability and efficacy of ipilimumab administered with or without prophylactic budesonide in patients with unresectable stage III or IV melanoma. Clin Cancer Res. (2009) 15(17):5591–8. 10.1158/1078-0432.CCR-09-1024 19671877

[62] Upadhrasta S Elias H Patel K Zheng L. Managing cardiotoxicity associated with immune checkpoint inhibitors. Chronic Dis Transl Med. (2019) 5(1):6–14. 10.1016/j.cdtm.2019.02.004 30993259 PMC6450824

[63] Aird WC. Endothelial cell heterogeneity. Cold Spring Harb Perspect Med. (2012) 2(1):a006429. 10.1101/cshperspect.a006429 22315715 PMC3253027

[64] Hsu PY Mammadova A Benkirane-Jessel N Desaubry L Nebigil CG. Updates on anticancer therapy-mediated vascular toxicity and new horizons in therapeutic strategies. Front Cardiovasc Med. (2021) 8:694711. 10.3389/fcvm.2021.694711 34386529 PMC8353082

[65] Daher IN Yeh ET. Vascular complications of selected cancer therapies. Nat Clin Pract Cardiovasc Med. (2008) 5(12):797–805. 10.1038/ncpcardio1375 18852710

[66] Claesson-Welsh L Dejana E McDonald DM. Permeability of the endothelial barrier: identifying and reconciling controversies. Trends Mol Med. (2021) 27(4):314–31. 10.1016/j.molmed.2020.11.006 33309601 PMC8005435

[67] Fernandez-Fernandez A Carvajal DA Lei T McGoron AJ. Chemotherapy-induced changes in cardiac capillary permeability measured by fluorescent multiple indicator dilution. Ann Biomed Eng. (2014) 42(12):2405–15. 10.1007/s10439-014-1110-9 25224075 PMC4241122

[68] Wolf MB Baynes JW. The anti-cancer drug, doxorubicin, causes oxidant stress-induced endothelial dysfunction. Biochim Biophys Acta. (2006) 1760(2):267–71. 10.1016/j.bbagen.2005.10.012 16337743

[69] Wilkinson EL Sidaway JE Cross MJ. Cardiotoxic drugs herceptin and doxorubicin inhibit cardiac microvascular endothelial cell barrier formation resulting in increased drug permeability. Biol Open. (2016) 5(10):1362–70. 10.1242/bio.020362 27543060 PMC5087671

[70] Wilkinson EL Sidaway JE Cross MJ. Statin regulated Erk5 stimulates tight junction formation and reduces permeability in human cardiac endothelial cells. J Cell Physiol. (2018) 233(1):186–200. 10.1002/jcp.26064 28639275 PMC5655747

[71] Elmore S. Apoptosis: a review of programmed cell death. Toxicol Pathol. (2007) 35(4):495–516. 10.1080/01926230701320337 17562483 PMC2117903

[72] Wu S Ko YS Teng MS Ko YL Hsu LA Hsueh C Adriamycin-induced cardiomyocyte and endothelial cell apoptosis: in vitro and in vivo studies. J Mol Cell Cardiol. (2002) 34(12):1595–607. 10.1006/jmcc.2002.2110 12505058

[73] Murata T Yamawaki H Yoshimoto R Hori M Sato K Ozaki H Chronic effect of doxorubicin on vascular endothelium assessed by organ culture study. Life Sci. (2001) 69(22):2685–95. 10.1016/s0024-3205(01)01352-2 11712671

[74] Rasanen M Degerman J Nissinen TA Miinalainen I Kerkela R Siltanen A Vegf-B gene therapy inhibits doxorubicin-induced cardiotoxicity by endothelial protection. Proc Natl Acad Sci USA. (2016) 113(46):13144–9. 10.1073/pnas.1616168113 27799559 PMC5135329

[75] Chow AY Chin C Dahl G Rosenthal DN. Anthracyclines cause endothelial injury in pediatric cancer patients: a pilot study. J Clin Oncol. (2006) 24(6):925–8. 10.1200/JCO.2005.03.5956 16484703

[76] Mikaelian I Buness A de Vera-Mudry MC Kanwal C Coluccio D Rasmussen E Primary endothelial damage is the mechanism of cardiotoxicity of tubulin-binding drugs. Toxicol Sci. (2010) 117(1):144–51. 10.1093/toxsci/kfq189 20624997

[77] Hemanthakumar KA Kivela R. Angiogenesis and angiocrines regulating heart growth. Vasc Biol. (2020) 2(1):R93–R104. 10.1530/VB-20-0006 32935078 PMC7487598

[78] Chiusa M Hool SL Truetsch P Djafarzadeh S Jakob SM Seifriz F Cancer therapy modulates vegf signaling and viability in adult rat cardiac microvascular endothelial cells and cardiomyocytes. J Mol Cell Cardiol. (2012) 52(5):1164–75. 10.1016/j.yjmcc.2012.01.022 22326847

[79] Graziani S Scorrano L Pontarin G. Transient exposure of endothelial cells to doxorubicin leads to long-lasting vascular endothelial growth factor receptor 2 downregulation. Cells. (2022) 11(2):210. 10.3390/cells11020210 PMC8773916 35053325

[80] Chaudhry R Miao JH Rehman A. Physiology, Cardiovascular. Treasure Island (FL): Statpearls (2022). 29630249

[81] Lemmey HAL Garland CJ Dora KA. Intrinsic regulation of microvascular tone by myoendothelial feedback circuits. Curr Top Membr. (2020) 85:327–55. 10.1016/bs.ctm.2020.01.004 32402644

[82] Chu TF Rupnick MA Kerkela R Dallabrida SM Zurakowski D Nguyen L Cardiotoxicity associated with tyrosine kinase inhibitor sunitinib. Lancet. (2007) 370(9604):2011–9. 10.1016/S0140-6736(07)61865-0 18083403 PMC2643085

[83] Iqubal A Iqubal MK Sharma S Ansari MA Najmi AK Ali SM Molecular mechanism involved in cyclophosphamide-induced cardiotoxicity: old drug with a new vision. Life Sci. (2019) 218:112–31. 10.1016/j.lfs.2018.12.018 30552952

[84] Chong JH Ghosh AK. Coronary artery vasospasm induced by 5-fluorouracil: proposed mechanisms, existing management options and future directions. Interv Cardiol. (2019) 14(2):89–94. 10.15420/icr.2019.12 31178935 PMC6545978

[85] de Vries MA Alipour A Birnie E Westzaan A van Santen S van der Zwan E Coronary leukocyte activation in relation to progression of coronary artery disease. Front Med. (2016) 10(1):85–90. 10.1007/s11684-016-0435-1 26831871

[86] Jones DP True HD Patel J. Leukocyte trafficking in cardiovascular disease: insights from experimental models. Mediators Inflamm. (2017) 2017:9746169. 10.1155/2017/9746169 28465628 PMC5390637

[87] Chen DY Huang WK Chien-Chia Wu V Chang WC Chen JS Chuang CK Cardiovascular toxicity of immune checkpoint inhibitors in cancer patients: a review when cardiology meets immuno-oncology. J Formos Med Assoc. (2020) 119(10):1461–75. 10.1016/j.jfma.2019.07.025 31444018

[88] Palaskas N Lopez-Mattei J Durand JB Iliescu C Deswal A. Immune checkpoint inhibitor myocarditis: pathophysiological characteristics, diagnosis, and treatment. J Am Heart Assoc. (2020) 9(2):e013757. 10.1161/JAHA.119.013757 31960755 PMC7033840

[89] Johnson DB Balko JM Compton ML Chalkias S Gorham J Xu Y Fulminant myocarditis with combination immune checkpoint blockade. N Engl J Med. (2016) 375(18):1749–55. 10.1056/NEJMoa1609214 27806233 PMC5247797

[90] Golebiewska EM Poole AW. Platelet secretion: from haemostasis to wound healing and beyond. Blood Rev. (2015) 29(3):153–62. 10.1016/j.blre.2014.10.003 25468720 PMC4452143

[91] Togna GI Togna AR Franconi M Caprino L. Cisplatin triggers platelet activation. Thromb Res. (2000) 99(5):503–9. 10.1016/s0049-3848(00)00294-2 10973681

[92] Kruger-Genge A Kohler S Laube M Haileka V Lemm S Majchrzak K Anti-cancer prodrug cyclophosphamide exerts thrombogenic effects on human venous endothelial cells independent of Cyp450 activation-relevance to thrombosis. Cells. (2023) 12(15):1965. 10.3390/cells12151965 37566045 PMC10416884

[93] Armulik A Genove G Betsholtz C. Pericytes: developmental, physiological, and pathological perspectives, problems, and promises. Dev Cell. (2011) 21(2):193–215. 10.1016/j.devcel.2011.07.001 21839917

[94] Chintalgattu V Rees ML Culver JC Goel A Jiffar T Zhang J Coronary microvascular pericytes are the cellular target of sunitinib malate-induced cardiotoxicity. Sci Transl Med. (2013) 5(187):187ra69. 10.1126/scitranslmed.3005066 23720580 PMC3833098

[95] Plana JC Galderisi M Barac A Ewer MS Ky B Scherrer-Crosbie M Expert consensus for multimodality imaging evaluation of adult patients during and after cancer therapy: a report from the American society of echocardiography and the European association of cardiovascular imaging. Eur Heart J Cardiovasc Imaging. (2014) 15(10):1063–93. 10.1093/ehjci/jeu192 25239940 PMC4402366

[96] Park KC Gaze DC Collinson PO Marber MS. Cardiac troponins: from myocardial infarction to chronic disease. Cardiovasc Res. (2017) 113(14):1708–18. 10.1093/cvr/cvx183 29016754 PMC5852618

[97] Kikuchi K Poss KD. Cardiac regenerative capacity and mechanisms. Annu Rev Cell Dev Biol. (2012) 28:719–41. 10.1146/annurev-cellbio-101011-155739 23057748 PMC3586268

[98] Bongiovanni C Sacchi F Da Pra S Pantano E Miano C Morelli MB Reawakening the intrinsic cardiac regenerative potential: molecular strategies to boost dedifferentiation and proliferation of endogenous cardiomyocytes. Front Cardiovasc Med. (2021) 8:750604. 10.3389/fcvm.2021.750604 34692797 PMC8531484

[99] Henriksen PA. Anthracycline cardiotoxicity: an update on mechanisms, monitoring and prevention. Heart. (2018) 104(12):971–7. 10.1136/heartjnl-2017-312103 29217634

[100] Ueno M Kakinuma Y Yuhki K Murakoshi N Iemitsu M Miyauchi T Doxorubicin induces apoptosis by activation of caspase-3 in cultured cardiomyocytes in vitro and rat cardiac ventricles in vivo. J Pharmacol Sci. (2006) 101(2):151–8. 10.1254/jphs.fp0050980 16766856

[101] Childs AC Phaneuf SL Dirks AJ Phillips T Leeuwenburgh C. Doxorubicin treatment in vivo causes cytochrome C release and cardiomyocyte apoptosis, as well as increased mitochondrial efficiency, superoxide dismutase activity, and Bcl-2:Bax ratio. Cancer Res. (2002) 62(16):4592–8. . 12183413

[102] Zhao L Zhang B. Doxorubicin induces cardiotoxicity through upregulation of death receptors mediated apoptosis in cardiomyocytes. Sci Rep. (2017) 7:44735. 10.1038/srep44735 28300219 PMC5353581

[103] Christidi E Brunham LR. Regulated cell death pathways in doxorubicin-induced cardiotoxicity. Cell Death Dis. (2021) 12(4):339. 10.1038/s41419-021-03614-x 33795647 PMC8017015

[104] Ma H Jones KR Guo R Xu P Shen Y Ren J. Cisplatin compromises myocardial Contractile function and mitochondrial ultrastructure: role of endoplasmic reticulum stress. Clin Exp Pharmacol Physiol. (2010) 37(4):460–5. 10.1111/j.1440-1681.2009.05323.x 19878217

[105] Refaie MMM Shehata S El-Hussieny M Abdelraheem WM Bayoumi AMA. Role of Atp-sensitive potassium channel (Katp) and Enos in mediating the protective effect of nicorandil in cyclophosphamide-induced cardiotoxicity. Cardiovasc Toxicol. (2020) 20(1):71–81. 10.1007/s12012-019-09535-8 31230218

[106] Mohan N Jiang J Dokmanovic M Wu WJ. Trastuzumab-mediated cardiotoxicity: current understanding, challenges, and frontiers. Antib Ther. (2018) 1(1):13–7. 10.1093/abt/tby003 30215054 PMC6131716

[107] Gordon LI Burke MA Singh AT Prachand S Lieberman ED Sun L Blockade of the Erbb2 receptor induces cardiomyocyte death through mitochondrial and reactive oxygen species-dependent pathways. J Biol Chem. (2009) 284(4):2080–7. 10.1074/jbc.M804570200 19017630 PMC2629107

[108] Grazette LP Boecker W Matsui T Semigran M Force TL Hajjar RJ Inhibition of Erbb2 causes mitochondrial dysfunction in cardiomyocytes: implications for herceptin-induced cardiomyopathy. J Am Coll Cardiol. (2004) 44(11):2231–8. 10.1016/j.jacc.2004.08.066 15582322

[109] Onitilo AA Engel JM Stankowski RV. Cardiovascular toxicity associated with adjuvant trastuzumab therapy: prevalence, patient characteristics, and risk factors. Ther Adv Drug Saf. (2014) 5(4):154–66. 10.1177/2042098614529603 25083270 PMC4110857

[110] Bouitbir J Alshaikhali A Panajatovic MV Abegg VF Paech F Krahenbuhl S. Mitochondrial oxidative stress plays a critical role in the cardiotoxicity of sunitinib: running title: sunitinib and oxidative stress in hearts. Toxicology. (2019) 426:152281. 10.1016/j.tox.2019.152281 31445075

[111] Frey N Katus HA Olson EN Hill JA. Hypertrophy of the heart: a new therapeutic target? Circulation. (2004) 109(13):1580–9. 10.1161/01.CIR.0000120390.68287.BB 15066961

[112] Sourdon J Facchin C Certain A Viel T Robin B Lager F Sunitinib-induced cardiac hypertrophy and the endothelin axis. Theranostics. (2021) 11(8):3830–8. 10.7150/thno.49837 33664864 PMC7914356

[113] Tschope C Ammirati E Bozkurt B Caforio ALP Cooper LT Felix SB Myocarditis and inflammatory cardiomyopathy: current evidence and future directions. Nat Rev Cardiol. (2021) 18(3):169–93. 10.1038/s41569-020-00435-x 33046850 PMC7548534

[114] Love VA Grabie N Duramad P Stavrakis G Sharpe A Lichtman A. Ctla-4 ablation and interleukin-12 driven differentiation synergistically augment cardiac pathogenicity of cytotoxic T lymphocytes. Circ Res. (2007) 101(3):248–57. 10.1161/CIRCRESAHA.106.147124 17569889

[115] Nishimura H Okazaki T Tanaka Y Nakatani K Hara M Matsumori A Autoimmune dilated cardiomyopathy in Pd-1 receptor-deficient mice. Science. (2001) 291(5502):319–22. 10.1126/science.291.5502.319 11209085

[116] Lucas JA Menke J Rabacal WA Schoen FJ Sharpe AH Kelley VR. Programmed death ligand 1 regulates a critical checkpoint for autoimmune myocarditis and pneumonitis in Mrl mice. J Immunol. (2008) 181(4):2513–21. 10.4049/jimmunol.181.4.2513 18684942 PMC2587295

[117] Wang J Okazaki IM Yoshida T Chikuma S Kato Y Nakaki F Pd-1 deficiency results in the development of fatal myocarditis in Mrl mice. Int Immunol. (2010) 22(6):443–52. 10.1093/intimm/dxq026 20410257

[118] Bhagat A Shrestha P Kleinerman ES. The innate immune system in cardiovascular diseases and its role in doxorubicin-induced cardiotoxicity. Int J Mol Sci. (2022) 23(23):14649. 10.3390/ijms232314649 PMC9739741 36498974

[119] Bhagat A Shrestha P Jeyabal P Peng Z Watowich SS Kleinerman ES. Doxorubicin-induced cardiotoxicity is mediated by neutrophils through release of neutrophil elastase. Front Oncol. (2022) 12:947604. 10.3389/fonc.2022.947604 36033503 PMC9400062

[120] Zhang H Xu A Sun X Yang Y Zhang L Bai H Self-maintenance of cardiac resident reparative macrophages attenuates doxorubicin-induced cardiomyopathy through the Sr-A1-C-Myc axis. Circ Res. (2020) 127(5):610–27. 10.1161/CIRCRESAHA.119.316428 32466726

[121] Kumari R Jat P. Mechanisms of cellular senescence: cell cycle arrest and senescence associated secretory phenotype. Front Cell Dev Biol. (2021) 9:645593. 10.3389/fcell.2021.645593 33855023 PMC8039141

[122] Shimizu I Minamino T. Cellular senescence in cardiac diseases. J Cardiol. (2019) 74(4):313–9. 10.1016/j.jjcc.2019.05.002 31202488

[123] Mitry MA Laurent D Keith BL Sira E Eisenberg CA Eisenberg LM Accelerated cardiomyocyte senescence contributes to late-onset doxorubicin-induced cardiotoxicity. Am J Physiol Cell Physiol. (2020) 318(2):C380–C91. 10.1152/ajpcell.00073.2019 31913702 PMC7052608

[124] Maejima Y Adachi S Ito H Hirao K Isobe M. Induction of premature senescence in cardiomyocytes by doxorubicin as a novel mechanism of myocardial damage. Aging Cell. (2008) 7(2):125–36. 10.1111/j.1474-9726.2007.00358.x 18031568

[125] Linders AN Dias IB Ovchinnikova ES Vermeer M Hoes MF Markousis Mavrogenis G Evaluation of senescence and its prevention in doxorubicin-induced cardiotoxicity using dynamic engineered heart tissues. JACC CardioOncol. (2023) 5(3):298–315. 10.1016/j.jaccao.2023.03.012 37397084 PMC10308053

[126] Booth LK Redgrave RE Folaranmi O Gill JH Richardson GD. Anthracycline-induced cardiotoxicity and senescence. Front Aging. (2022) 3:1058435. 10.3389/fragi.2022.1058435 36452034 PMC9701822

[127] Tallquist MD. Cardiac fibroblasts: from origin to injury. Curr Opin Physiol. (2018) 1:75–9. 10.1016/j.cophys.2017.08.002 29527587 PMC5839664

[128] Zhan H Aizawa K Sun J Tomida S Otsu K Conway SJ Ataxia telangiectasia mutated in cardiac fibroblasts regulates doxorubicin-induced cardiotoxicity. Cardiovasc Res. (2016) 110(1):85–95. 10.1093/cvr/cvw032 26862121 PMC4798048

[129] Cappetta D Esposito G Piegari E Russo R Ciuffreda LP Rivellino A Sirt1 activation attenuates diastolic dysfunction by reducing cardiac fibrosis in a model of anthracycline cardiomyopathy. Int J Cardiol. (2016) 205:99–110. 10.1016/j.ijcard.2015.12.008 26730840

[130] Levick SP Soto-Pantoja DR Bi J Hundley WG Widiapradja A Manteufel EJ Doxorubicin-induced myocardial fibrosis involves the neurokinin-1 receptor and direct effects on cardiac fibroblasts. Heart Lung Circ. (2019) 28(10):1598–605. 10.1016/j.hlc.2018.08.003 30205930 PMC7901001

[131] Narikawa M Umemura M Tanaka R Hikichi M Nagasako A Fujita T Doxorubicin induces trans-differentiation and Mmp1 expression in cardiac fibroblasts via cell death-independent pathways. PLoS One. (2019) 14(9):e0221940. 10.1371/journal.pone.0221940 31513610 PMC6742217

[132] Collins TA Rolf MG Pointon A. Current and future approaches to nonclinical cardiovascular safety assessment. Drug Discov Today. (2020) 25(7):1129–34. 10.1016/j.drudis.2020.03.011 32209428

[133] Gintant G Sager PT Stockbridge N. Evolution of strategies to improve preclinical cardiac safety testing. Nat Rev Drug Discov. (2016) 15(7):457–71. 10.1038/nrd.2015.34 26893184

[134] Morton MJ Armstrong D Abi Gerges N Bridgland-Taylor M Pollard CE Bowes J Predicting changes in cardiac myocyte contractility during early drug discovery with in vitro assays. Toxicol Appl Pharmacol. (2014) 279(2):87–94. 10.1016/j.taap.2014.06.005 24952337

[135] Ravenscroft SM Pointon A Williams AW Cross MJ Sidaway JE. Cardiac non-myocyte cells show enhanced pharmacological function suggestive of contractile maturity in stem cell derived cardiomyocyte microtissues. Toxicol Sci. (2016) 152(1):99–112. 10.1093/toxsci/kfw069 27125969 PMC4922542

[136] Giacomelli E Meraviglia V Campostrini G Cochrane A Cao X van Helden RWJ Human-Ipsc-derived cardiac stromal cells enhance maturation in 3d cardiac microtissues and reveal non-cardiomyocyte contributions to heart disease. Cell Stem Cell. (2020) 26(6):862–79 e11. 10.1016/j.stem.2020.05.004 32459996 PMC7284308

[137] Campostrini G Windt LM van Meer BJ Bellin M Mummery CL. Cardiac tissues from stem cells: new routes to maturation and cardiac regeneration. Circ Res. (2021) 128(6):775–801. 10.1161/CIRCRESAHA.121.318183 33734815 PMC8410091

[138] Arslan U Moruzzi A Nowacka J Mummery CL Eckardt D Loskill P Microphysiological stem cell models of the human heart. Mater Today Bio. (2022) 14:100259. 10.1016/j.mtbio.2022.100259 35514437 PMC9062349

[139] Ergir E Oliver-De La Cruz J Fernandes S Cassani M Niro F Pereira-Sousa D Generation and maturation of human Ipsc-derived 3d organotypic cardiac microtissues in long-term culture. Sci Rep. (2022) 12(1):17409. 10.1038/s41598-022-22225-w 36257968 PMC9579206

[140] Archer CR Sargeant R Basak J Pilling J Barnes JR Pointon A. Characterization and validation of a human 3d cardiac microtissue for the assessment of changes in cardiac pathology. Sci Rep. (2018) 8(1):10160. 10.1038/s41598-018-28393-y 29976997 PMC6033897

[141] Burnham MP Harvey R Sargeant R Fertig N Haddrick M. A scalable approach reveals functional responses of Ipsc cardiomyocyte 3d spheroids. SLAS Discov. (2021) 26(3):352–63. 10.1177/2472555220975332 33283596

